# Effects of Age Category and eCG Dose on Reproductive Performance in Ile-de-France Ewe Lambs and Adult Ewes Under Seasonal-Transition Field Conditions

**DOI:** 10.3390/vetsci13090992

**Published:** 2026-09-19

**Authors:** Muhammed Furkan Ciftci, Fatma Satilmis

**Affiliations:** Department of Obstetrics and Gynecology, Faculty of Veterinary Medicine, Selcuk University, Konya 42130, Türkiye; mf.cftc@gmail.com

**Keywords:** adult ewe, eCG dose, ewe lamb, Ile-de-France, oestrus synchronization, reproductive performance

## Abstract

This field study evaluated the effects of 400 and 500 IU equine chorionic gonadotropin (eCG) on oestrus response, fertility, and lambing performance in Ile-de-France ewe lambs and adult ewes under seasonal-transition field conditions. A total of 914 animals were assigned to four groups according to age category and eCG dose: ewe lambs receiving 400 IU (EL400, *n* = 173) or 500 IU eCG (EL500, *n* = 169), and adult ewes receiving 400 IU (AE400, *n* = 301) or 500 IU eCG (AE500, *n* = 271), following a 7-day intravaginal progesterone-based synchronization protocol. Oestrus rate did not differ among groups (*p* = 0.861), indicating that both doses were similarly effective in inducing behavioural oestrus, whereas the interval to oestrus onset showed significant main effects of both age category (*p* < 0.001) and eCG dose (*p* < 0.001), with no significant age × dose interaction (*p* = 0.559). Using a 2 × 2 factorial analysis, significant main effects of age category were observed for outcomes including pregnancy rate following oestrus, overall pregnancy rate, and fecundity (*p* = 0.001, *p* = 0.009, and *p* = 0.004, respectively), with higher values in adult ewes than in ewe lambs regardless of eCG dose. In contrast, 500 IU eCG was associated with a greater overall litter size and multiple birth rate than 400 IU eCG (*p* = 0.039 and *p* = 0.033, respectively). The age × dose interactions for litter size and multiple birth rate were not statistically significant (*p* = 0.058 and *p* = 0.071, respectively). The age × dose interaction for lambing rate was also not statistically significant (*p* = 0.064). These results indicate that age category and eCG dose were associated with different aspects of reproductive performance. Under the specific seasonal-transition field conditions of this single-breed, single-farm study, selecting the eCG dose according to the reproductive objective may represent a practical management consideration.

## Simple Summary

Managing sheep reproduction during seasonal-transition periods often requires tailored approaches under practical field conditions. This study evaluated whether 400 IU or 500 IU of eCG optimized reproductive outcomes in Ile-de-France ewe lambs and adult ewes during a seasonal-transition period. A total of 914 animals received a standard synchronization program followed by one of the two eCG doses. Both doses were similarly effective in inducing oestrus. However, adult ewes achieved higher pregnancy-related outcomes than younger ewe lambs, regardless of the eCG dose used. Overall, the higher eCG dose (500 IU) was associated with a greater proportion of multiple births and a larger litter size, while the corresponding age × dose interactions were not statistically significant. Under the specific seasonal-transition field conditions of this single-farm study, these findings suggest that age category and eCG dose were associated with different aspects of reproductive performance.

## 1. Introduction

Sheep farming is an important branch of animal agriculture worldwide in terms of meat, milk, and wool production, and reproductive performance plays a decisive role in the economic sustainability of sheep enterprises [1]. In sustainable sheep production systems, lamb output and lambing rates are among the main indicators of productivity and directly affect farm profitability. Therefore, improving and controlling reproductive performance in sheep has become one of the primary goals of modern production systems [2].

Reproductive activity in sheep is influenced by photoperiod, breed, nutrition, management, and environmental conditions. In many sheep breeds, changes in day length affect the hypothalamic–pituitary–ovarian axis and contribute to seasonal variation in ovarian activity [3,4]. However, the degree of seasonality is not uniform among breeds, and some breeds may maintain reproductive activity across a wider part of the year under favorable management and nutritional conditions. Therefore, reproductive responses to synchronization protocols should be interpreted within the specific breed, physiological status, and field conditions in which they are applied [5,6]. In this context, progesterone-based oestrus synchronization protocols combined with gonadotropin treatment are widely used to improve reproductive management, concentrate mating and lambing, and obtain more predictable reproductive outcomes under field conditions [6,7,8].

Seasonal-transition periods may represent a practical challenge for flock reproductive management because reproductive responses to synchronization protocols can become more variable depending on breed, age, physiological status, nutrition, and environmental conditions. In Ile-de-France ewes, variations in LH secretion and ovarian responsiveness can occur during seasonal-transition periods. Such variability under practical field conditions necessitates the evaluation of different eCG doses to achieve predictable and optimal reproductive outcomes [9,10].

Equine chorionic gonadotropin (eCG) is a hormone widely used in oestrus synchronization protocols in sheep because it stimulates follicular development and increases the ovulation rate. However, it has been reported that the dose of eCG may influence not only the occurrence of oestrus, but also fertility parameters, embryonic development, and birth type [11,12,13]. Although higher doses of eCG may enhance oestrus induction and ovulation rates, they do not always result in a parallel improvement in fertilisation and pregnancy rates and may even increase embryonic losses [14].

Another important factor affecting reproductive performance in sheep is the age and physiological status of the animal. Significant differences may exist between ewe lambs and adult ewes in terms of ovarian activity, hormonal response, and fertility [15]. Ewe lambs generally have lower ovulation rates, and their response to hormonal treatments may be more variable than that of adult ewes [16]. However, studies evaluating the effects of different eCG doses across these distinct physiological groups are limited. Therefore, it is important to investigate this relationship in different age groups.

The Ile-de-France is a meat breed characterized by favourable growth and carcass traits and widely used in crossbreeding programmes, while also exhibiting satisfactory reproductive performance [17,18,19,20,21]. Although eCG-based synchronization has been evaluated in adult Ile-de-France ewes [22], direct comparisons of eCG dose responses between ewe lambs and adult ewes remain limited. Physiological maturity and environmental factors can influence the ovarian response to eCG-based synchronization protocols. Previous studies have shown that the reproductive response to different eCG doses may vary among sheep populations and physiological categories, and that increasing the dose does not consistently lead to proportional improvements in fertility-related outcomes [14,23]. These inconsistent responses suggest that findings obtained from one breed or physiological category should not be directly extrapolated to another. Existing studies have demonstrated that environmental factors, such as photoperiod and nutrition, cause changes in LH secretion and ovarian activity in Ile-de-France ewes [24]. Furthermore, endocrine studies in this breed have documented variation in LH secretion and ovarian cyclicity depending on these external factors [10,24,25]. In this context, eCG administered at progestogen withdrawal is commonly used to support follicular development, while ovarian responses to eCG may vary according to season and physiological category [14,23,26]. Therefore, comparing 400 and 500 IU eCG in ewe lambs and adult ewes provides a practical framework for determining whether a modest increase in dose is associated with different reproductive responses between age categories under seasonal-transition field conditions.

The present study was designed to evaluate age-related reproductive responses to two different eCG doses in Ile-de-France ewe lambs and adult ewes under Northern Hemisphere seasonal-transition field conditions. For this purpose, oestrus response, fertility, and lambing performance were evaluated among the study groups. We hypothesized that both age category and eCG dose would be associated with reproductive performance and that the response to eCG dose might differ between ewe lambs and adult ewes.

## 2. Materials and Methods

### 2.1. Study Location and Period

The present study was carried out at a private farm raising Ile-de-France sheep in Konya, Türkiye (38°11′26″ N, 32°22′25″ E). The research was conducted during the winter-to-spring seasonal-transition period (January–April) under Northern Hemisphere conditions.

### 2.2. Animals, Experimental Design, and Management

All animals included in the study belonged to the Ile-de-France breed. All females and rams used in the study originated from the same farm. At the beginning of the experiment, the body condition scores (BCS) of the animals ranged from 2.5 to 3.5 on a standard 5-point scale. BCS was used as an inclusion criterion within the predefined range of 2.5–3.5 and was not evaluated as an independent study variable. The animal material of the study consisted of adult ewes aged 2–4 years (70–90 kg live body weight) and nulliparous ewe lambs aged 11–14 months (50–60 kg live body weight). The adult ewe group consisted of animals that had lambed previously, including both primiparous and multiparous ewes. The interval between previous lambing and the start of synchronization was not systematically recorded and was therefore not included as a study variable. They were not managed for milk production, and no milking was performed; lambs were allowed to suckle naturally during lactation. The ewe lamb group consisted of nulliparous females that had previously exhibited behavioral oestrus, as documented by routine farm observations and breeding records. The rams used for natural mating were selected based on farm records indicating high mating success and satisfactory pregnancy rates during the previous breeding season. Only clinically healthy animals were included in the study. Animals showing any signs of systemic disease on general examination, as well as those with lameness, malodorous vaginal discharge, or any genital abnormalities detected on external inspection, were excluded. After clinical screening, the eligible animals were assigned under farm conditions to four study groups according to age category and planned eCG dose. Within the adult ewe category, animals were stratified according to parity (primiparous or multiparous) and were then randomly allocated by computer to the 400 or 500 IU eCG groups to obtain similar parity distributions between the two treatment groups. Parity was therefore used as a stratification variable to balance the adult ewe treatment groups and was not evaluated as an independent experimental factor. Ewe lambs, all of which were nulliparous, were randomly allocated by computer to the two eCG dose groups. Accordingly, the animals were classified as follows: ewe lambs treated with 400 IU eCG (EL400, *n* = 173), ewe lambs treated with 500 IU eCG (EL500, *n* = 169), adult ewes treated with 400 IU eCG (AE400, *n* = 301), and adult ewes treated with 500 IU eCG (AE500, *n* = 271). The group sizes reported represent the animals included in the final analysis. Animals that left the flock during the study for routine farm-related reasons unrelated to the experimental treatments, such as sale or death, were excluded from all analyses, resulting in unequal final group sizes.

Throughout the study period, all animals were maintained under similar environmental and management conditions and were fed a standard ration formulated to meet maintenance requirements. The ration consisted of wheat, corn, corn silage, and roughage. Ewes diagnosed with multiple gestation received additional energy-dense feed during the last trimester of pregnancy. Feeding was provided twice daily, in the morning and evening, and water was available ad libitum.

### 2.3. Oestrus Synchronization Protocol

A progesterone-based oestrus synchronization protocol was applied to all animals. For this purpose, an intravaginal sponge containing 20 mg fluorogestone acetate (Chronogest CR^®^, MSD Animal Health, Igoville, France) was inserted intravaginally. The sponges were left in the vagina for 7 days and were removed at the end of Day 7. Although the conventional recommended duration of Chronogest CR^®^ treatment is 12–14 days, a 7-day short-term protocol was selected based on previously reported short-term FGA protocols in sheep, including Ile-de-France ewes. Following sponge removal, animals in the EL400 and AE400 groups received 400 IU eCG (Chronogest^®^ PMSG 6000 IU, MSD Animal Health, Unterschleissheim, Germany), whereas those in the EL500 and AE500 groups received 500 IU eCG intramuscularly.

### 2.4. Mating Procedure and Oestrus Detection

At 24 h after eCG administration, rams with known fertility and proven mating ability were introduced for natural mating. Mating was carried out in paddocks with similar physical and management characteristics. Different rams were randomly assigned to each experimental group, with no ram used in more than one group, and the same ram-to-ewe ratio (1:8) was maintained across all groups. The rams remained with the animals for 7 days. Because mating occurred naturally in group paddocks, individual ewe-to-ram mating assignments were not available for analysis; therefore, ram-level effects could not be included in the statistical model.

For oestrus detection, the rams were fitted with marking harnesses, a practical method widely used in sheep [7]. Following eCG administration, the animals were monitored at approximately 1- to 2-h intervals throughout the day for signs of oestrus, and those marked by the rams were considered to have exhibited oestrus. The interval to oestrus onset was recorded as the time from sponge removal to the first observed marking. Personnel responsible for oestrus recording were not informed of the treatment-group assignments.

### 2.5. Pregnancy Diagnosis

**Figure 1:**
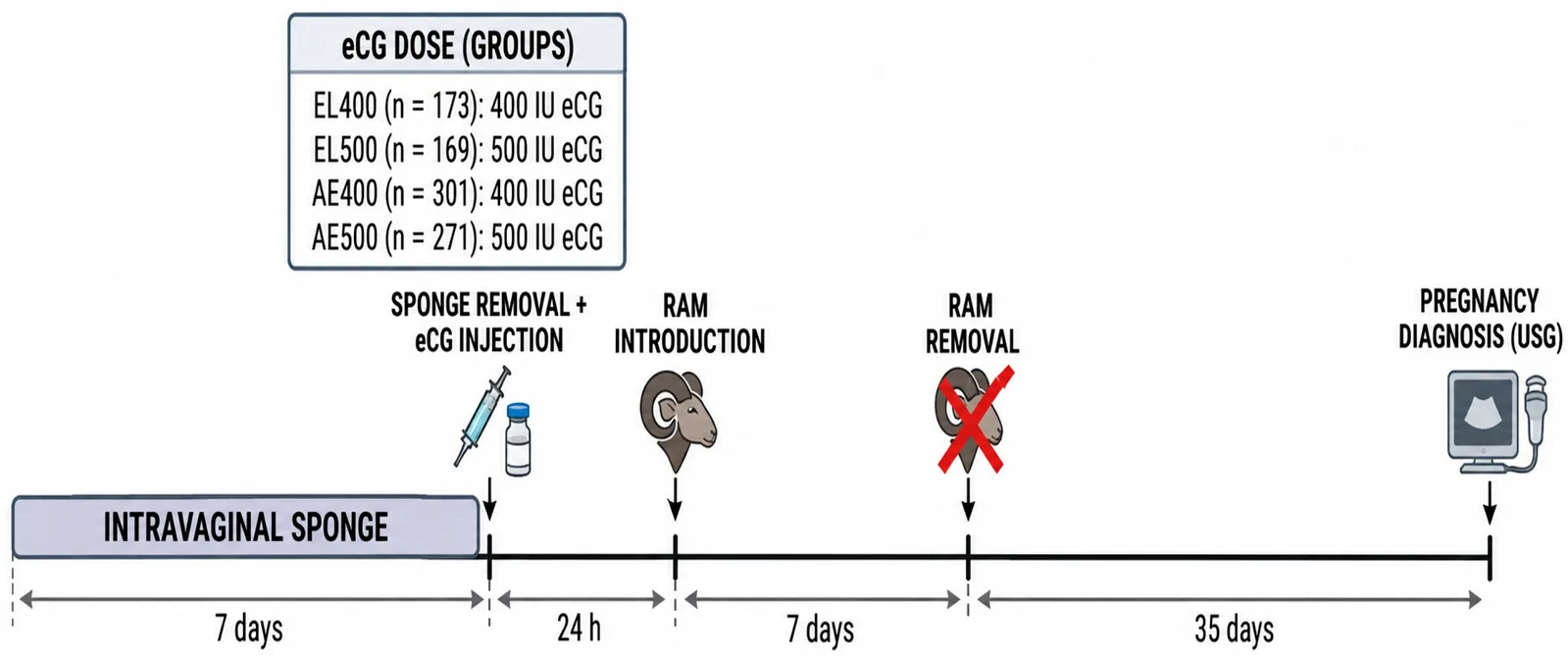
Experimental design and synchronization protocol of ewe lambs and adult ewes treated with 400 or 500 IU eCG. EL400 and EL500 represent ewe lambs treated with 400 and 500 IU eCG, respectively; AE400 and AE500 represent adult ewes treated with 400 and 500 IU eCG, respectively. The intravaginal sponge was removed after 7 days, and eCG was administered at sponge removal. Rams were introduced 24 h later and remained with the females for 7 days. Pregnancy diagnosis was performed by ultrasonography 35 days after ram removal.

Pregnancy examinations were performed transabdominally on Day 35 after ram removal using a real-time ultrasonography device (SIUI 2100, SIUI, Shantou, China) equipped with a 5 MHz probe. Ultrasonographic pregnancy examinations were performed by an investigator who was aware of the treatment-group assignments. Visualization of a fetus within an anechoic area in the uterus was considered a positive pregnancy diagnosis, whereas the presence of more than one fetus was regarded as multiple pregnancy. The accuracy of Day 35 transabdominal ultrasonography for fetal number determination was not specifically validated in this flock; however, single and multiple birth outcomes were classified according to the actual number of lambs born at parturition rather than the ultrasonographic fetal count. All animals diagnosed as pregnant were followed until parturition, and the number of lambs born and birth type (single or multiple) were recorded. Perinatal lamb loss, defined as lambs born dead or dying within the first 24 h after birth, was assessed from the available parturition and farm records. The experimental design of the study is presented in Figure 1.

### 2.6. Reproductive Parameters Evaluated

In the study, the following parameters were used to evaluate oestrus response, fertility, and lambing performance; their calculation and unit of expression are summarized in Table 1. Lambs born dead were included in the total number of lambs born. Lambs born dead and lambs dying within the first 24 h after birth were recorded together as perinatal lamb loss; these two categories were not distinguished in the farm records.

### 2.7. Statistical Analysis

Statistical analysis of the obtained data was performed using SPSS 27.0 (IBM SPSS Statistics for Windows, Version 27.0; IBM Corp., Armonk, NY, USA). The normality of continuous data was assessed using the Shapiro–Wilk test, and homogeneity of variances was evaluated with Levene’s test. Because the four experimental groups reflect a 2 × 2 factorial design defined by age category (ewe lamb and adult ewe) and eCG dose (400 and 500 IU eCG), continuous variables were analyzed using two-way analysis of variance (ANOVA), with age category, eCG dose, and their interaction as fixed effects. Differences in proportional data among the four groups were evaluated using the chi-square test, and the effects of age category, eCG dose, and their interaction were further analyzed using factorial logistic regression. Because lambing rate and pregnancy loss after pregnancy diagnosis are mathematically complementary outcomes based on the same denominator, only lambing rate was included in the inferential analyses, whereas pregnancy loss was reported descriptively as the complementary outcome. Perinatal lamb loss was analysed at the lamb level. Given the small number of non-lambing events, the *p*-value for the age × dose interaction in lambing rate was additionally evaluated using a Monte Carlo permutation test implemented in Python (v3.12.3; NumPy v2.4.4); the corresponding OR and 95% CI were obtained from the standard factorial logistic regression model. Effect estimates with 95% confidence intervals (CIs) were obtained from the corresponding factorial models, while for the age × dose interaction in lambing rate, only the *p*-value was obtained from the Monte Carlo permutation test described above. Effect estimates are reported as odds ratios (ORs) for binary outcomes and mean differences (Δ) for continuous outcomes. For the binary outcomes, odds ratios were derived from factorial logistic regression models including both age category and eCG dose, so that the reported effect of each factor was adjusted for the other factor. Primary analyses were conducted without adjustment for multiple testing across the nine reproductive outcomes analysed. As a sensitivity analysis, the Benjamini–Hochberg false discovery rate (FDR) procedure was applied jointly to all 27 hypothesis tests (9 outcomes × 3 factorial effects: age category, eCG dose, and age × dose interaction), treating them as a single family. Continuous data were expressed as mean ± standard error of the mean (SEM). Statistical significance was accepted at *p* < 0.05.

## 3. Results

**Figure 2:**
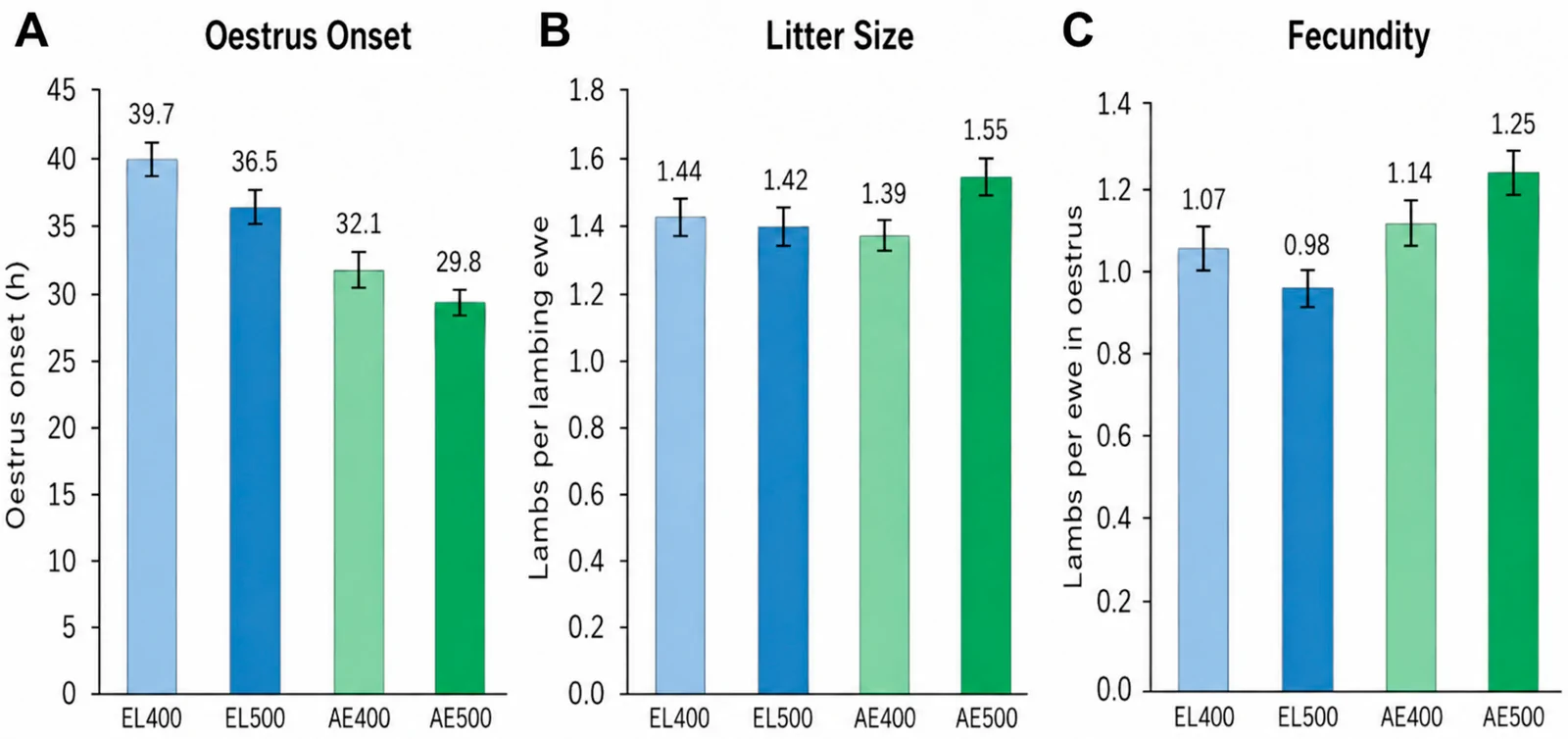
(**A**) Oestrus onset; (**B**) litter size; (**C**) fecundity in ewe lambs and adult ewes treated with 400 or 500 IU eCG. Values are presented as mean ± SEM. Sample sizes for oestrus onset and fecundity were EL400, *n* = 128; EL500, *n* = 129; AE400, *n* = 225; and AE500, *n* = 209; for litter size, the corresponding sample sizes were 95, 89, 185, and 169. Factorial analysis showed significant main effects of age category and eCG dose on oestrus onset, eCG dose on litter size, and age category on fecundity. However, the numerical dose-related patterns for litter size and fecundity differed between age categories, while the corresponding age × dose interactions were not statistically significant. EL400 and EL500 represent ewe lambs treated with 400 and 500 IU eCG, respectively; AE400 and AE500 represent adult ewes treated with 400 and 500 IU eCG, respectively.

Oestrus rate did not differ significantly among the experimental groups (*p* = 0.861). No significant effect of age category (*p* = 0.792), eCG dose (*p* = 0.406), or their interaction (*p* = 0.989) was detected for oestrus rate. In contrast, the interval to oestrus onset differed significantly among the groups (*p* < 0.001; Figure 2A). The mean interval to oestrus onset was longer in the ewe lamb groups and shorter in the adult ewe groups. This interval was 39.7 ± 0.6 h and 36.5 ± 0.4 h in the EL400 and EL500 groups, respectively, whereas it was 32.1 ± 0.9 h and 29.8 ± 0.7 h in the AE400 and AE500 groups, respectively. Factorial analysis showed significant main effects of both age category (*p* < 0.001) and eCG dose (*p* < 0.001) on the interval to oestrus onset, with no significant age × dose interaction (*p* = 0.559). Adult ewes reached oestrus earlier than ewe lambs regardless of dose, and animals receiving 500 IU reached oestrus earlier than those receiving 400 IU overall, with no significant age × dose interaction.

Pregnancy rate following oestrus differed significantly among the experimental groups (*p* = 0.002). Factorial analysis showed a significant main effect of age category on pregnancy rate following oestrus (*p* = 0.001), with higher rates in adult ewes than in ewe lambs, while neither eCG dose (*p* = 0.547) nor the age × dose interaction (*p* = 0.186) was significant. Overall pregnancy rate also differed significantly among the experimental groups (*p* = 0.046). Similarly, factorial analysis showed a significant main effect of age category on overall pregnancy rate (*p* = 0.009), with no significant effect of eCG dose (*p* = 0.870) or age × dose interaction (*p* = 0.281).

Lambing rate did not differ significantly among the groups in the omnibus test (*p* = 0.157). No significant main effect of age category (*p* = 0.684) or eCG dose (*p* = 0.379) was detected, and the age × dose interaction was also not statistically significant (*p* = 0.064). Numerically, the 500 IU group showed a higher lambing rate than the 400 IU group among ewe lambs, whereas the opposite pattern was observed among adult ewes. Given the small number of non-lambing animals in each group (4, 1, 3, and 9, respectively), these numerical patterns should be interpreted with caution. Pregnancy loss after pregnancy diagnosis was reported descriptively as the complementary outcome to lambing rate (Table 2).

Single and multiple birth rates among the experimental groups were statistically significant (*p* = 0.049 for both). Factorial analysis showed a significant main effect of eCG dose on multiple birth rate (*p* = 0.033), with a higher overall rate in animals receiving 500 IU than in those receiving 400 IU (47.67% vs. 38.57%, respectively). However, the numerical pattern differed between age categories: multiple birth rate was higher with 500 IU in adult ewes, whereas a slight numerical decrease was observed in ewe lambs. Neither age category (*p* = 0.843) nor the age × dose interaction (*p* = 0.071) was significant.

Stillbirth and death within the first 24 h after birth were recorded together as perinatal lamb loss, as these two categories were not distinguished in the farm records. Overall, 91.18% (713/782) of lambs born were alive at 24 h; group-specific values are presented in Table 2 as the mathematical complement of perinatal lamb loss. Perinatal lamb loss did not differ significantly among the experimental groups (*p* = 0.210). Factorial analysis showed a significant main effect of age category (*p* = 0.039), with a lower perinatal lamb loss in adult ewes than in ewe lambs, whereas no significant effect of eCG dose (*p* = 0.993) or age × dose interaction (*p* = 0.670) was detected. The findings related to the reproductive parameters evaluated in this study are presented in detail in Table 2, while the effect estimates with 95% confidence intervals and *p*-values from the 2 × 2 factorial analyses of age category, eCG dose, and their interaction are summarized in Table 3.

For ease of interpretation, the odds ratios with 95% confidence intervals for the binary outcomes showing significant single main effects are summarized separately in Table 4. Adult ewes had 1.44 times higher odds of achieving pregnancy overall (95% CI: 1.09–1.89) and 1.94 times higher odds of becoming pregnant following oestrus (95% CI: 1.33–2.83) compared with ewe lambs. Animals receiving 500 IU eCG had 1.45 times higher odds of multiple birth compared with those receiving 400 IU (95% CI: 1.03–2.04). Lambs born to adult ewes had 0.59 times the odds of perinatal loss compared with those born to ewe lambs (95% CI: 0.36–0.97).

As presented in Figure 2, litter size differed significantly among the experimental groups (*p* = 0.038; Figure 2B). The mean litter size values in the EL400, EL500, AE400, and AE500 groups were 1.44 ± 0.05, 1.42 ± 0.05, 1.39 ± 0.04, and 1.55 ± 0.04, respectively. Factorial analysis showed a significant main effect of eCG dose on litter size (*p* = 0.039), with no significant main effect of age category (*p* = 0.448) or age × dose interaction (*p* = 0.058). Numerically, litter size increased with 500 IU in adult ewes but showed a slight decrease in ewe lambs; however, the age × dose interaction was not statistically significant. In contrast, fecundity differed significantly among the groups (*p* = 0.010; Figure 2C). The mean fecundity values were 1.07 ± 0.07, 0.98 ± 0.07, 1.14 ± 0.05, and 1.25 ± 0.06 in the EL400, EL500, AE400, and AE500 groups, respectively. Factorial analysis showed a significant main effect of age category on fecundity (*p* = 0.004), with no significant effect of eCG dose (*p* = 0.548) or age × dose interaction (*p* = 0.090).

## 4. Discussion

Reproductive responses to synchronization protocols in sheep may vary according to gonadotropin dose and the reproductive maturity of the animals. In the present study, the effects of two eCG doses on reproductive performance were evaluated in Ile-de-France ewe lambs and adult ewes under seasonal-transition field conditions. Although the initial oestrus response was similar across groups, subsequent reproductive outcomes differed according to age category and eCG dose. These findings indicate that age category and eCG dose were associated with different reproductive outcomes under the field conditions of this study.

The similar oestrus rates observed among the groups indicate that both 400 IU and 500 IU eCG were associated with comparable behavioural oestrus responses when used in combination with the progesterone-based synchronization protocol. In sheep, eCG is known to promote follicular development and support ovulatory capacity through its prolonged gonadotropic activity [27]. However, increasing the dose beyond a certain range does not always result in a higher oestrus rate, and particularly under seasonal-transition or variable field conditions, the effect of dose escalation on the incidence of oestrus may remain limited [28,29].

The finding that the interval to oestrus onset varied according to age and dose indicates that this parameter may be more sensitive than oestrus rate in reflecting differences in the response to treatment. The longer interval to oestrus onset observed in ewe lambs is consistent with previous reports describing later or less distinct expression of oestrous behaviour in young sheep [30]. Altınçekiç et al. (2018) [31] likewise reported that eCG dose affected the timing of oestrus onset in sheep. In the present study, the interval to oestrus onset was shorter with 500 IU eCG in both age groups. Consistent with this finding, several studies have shown that different eCG doses can influence the timing of oestrus onset, although this relationship is not always linear [32].

The significant difference observed in pregnancy rate following oestrus among the groups, together with the generally higher values recorded in adult ewes, indicates that fertility differed according to age category. Previous studies have likewise reported lower reproductive performance in ewe lambs than in adult ewes [30,33], while age-related differences in early-pregnancy progesterone status have also been described [34]. These factors were not evaluated in the present study. This age-related pattern is further supported by the 2 × 2 factorial analysis, which showed a significant main effect of age category on pregnancy rate following oestrus, with no significant effect of eCG dose or age × dose interaction.

The higher overall pregnancy rates observed in adult ewes likewise indicate that the difference seen in pregnancy rate following oestrus was also reflected at the stage of pregnancy establishment. Lower fertility in ewe lambs than in adult ewes has also been reported previously and has been associated with age-related physiological maturity [15,35,36]. Consistent with this, factorial analysis showed a significant main effect of age category on overall pregnancy rate, while no significant effect of eCG dose or age × dose interaction was detected. Therefore, although oestrus rates were similar in the present study, the lower pregnancy rates observed in ewe lambs may be considered a biologically expected outcome [9,30].

Lambing rates were high across all treatment groups, and the age × dose interaction was not statistically significant (*p* = 0.064). Numerically, the 500 IU group showed a higher lambing rate than the 400 IU group among ewe lambs, whereas the opposite pattern was observed among adult ewes. Pregnancy loss after pregnancy diagnosis is mathematically complementary to lambing rate and therefore reflected the same numerical pattern; accordingly, it was reported descriptively rather than treated as an independent inferential outcome. In ruminants, most pregnancy losses occur during the first trimester, whereas losses after pregnancy diagnosis, once maternal recognition of pregnancy has been completed, are generally less frequent [37,38,39,40]. Given the small number of non-lambing animals, these numerical age-specific patterns should be interpreted cautiously and should not be considered evidence of differential dose responsiveness.

Multiple birth rate showed a significant main effect of eCG dose, with a higher rate in animals receiving 500 IU than in those receiving 400 IU when pooled across age categories. This finding is consistent with previous reports showing an association between higher eCG doses and increased multiple ovulation or litter size in sheep [24,32,41,42,43]. Because ovulation rate was not directly measured in the present study, the mechanism underlying the observed dose-related difference in multiple birth rate cannot be determined from these data.

Litter size likewise showed a significant main effect of eCG dose, paralleling the pattern observed for multiple birth rate and consistent with previous reports of progestagen–eCG protocols in sheep [44]. In contrast, the higher fecundity observed in adult ewes was associated with a significant main effect of age category. This finding is consistent with the higher pregnancy rates observed in adult ewes rather than with a direct dose effect on prolificacy. Fecundity depends not only on ovulation rate but also on pregnancy establishment, maintenance of pregnancy, and the number of lambs born at parturition [30,45]. Although the 500 IU dose was associated with a higher multiple birth rate and a larger litter size, perinatal lamb loss was comparable between animals receiving 500 IU and 400 IU eCG (8.76% vs. 8.88%, respectively). Under the present management conditions, the higher prolificacy associated with 500 IU eCG was therefore not accompanied by an increase in early lamb loss. Perinatal lamb loss was, however, lower in adult ewes than in ewe lambs, consistent with the age-related pattern observed for the other reproductive outcomes.

Overall, in the primary factorial analyses, eCG dose was statistically associated with the interval to oestrus onset, multiple birth rate, and litter size. No significant association with eCG dose was detected for oestrus rate, overall pregnancy rate, pregnancy rate following oestrus, lambing rate, fecundity, or perinatal lamb loss. Age category was statistically associated with oestrus onset, overall pregnancy rate, pregnancy rate following oestrus, fecundity, and perinatal lamb loss. No significant age × dose interaction was detected for the reproductive outcomes associated with eCG dose. Thus, age- and dose-related associations involved different reproductive outcomes under the present field conditions.

When interpreting the present findings, it should be considered that the field design did not include an untreated control group. In studies conducted outside the main breeding season or under seasonal-transition conditions, spontaneous reproductive activity in untreated ewes has generally been lower than the responses obtained after hormonal synchronization, although reported values vary according to breed, physiological status, and season. Berean et al. (2021) [46] reported an oestrus rate of 6.25% and a pregnancy rate of 4.16% in untreated Lacaune ewes, whereas the treated groups had higher values, with an oestrus rate of 100% and pregnancy rates ranging from 89.58% to 95.83%. Martinez et al. (2015) [47] also reported lower ovarian activity in untreated ewe lambs than in synchronized animals, with ovulation rates of 3.2% and 95.4%, respectively. These data provide useful context for the relatively high oestrus and pregnancy rates observed in the present study; however, because an untreated control group was not included, the extent to which spontaneous reproductive activity may have contributed to these responses under the present field conditions cannot be directly determined. Accordingly, in the absence of a concurrent untreated control, the observed age- and dose-related differences should be interpreted as associations among treated groups rather than as effects causally attributable solely to eCG, because spontaneous reproductive activity during the seasonal-transition period may also vary with age category.

Although the present study included a large field-based population and provides practical reproductive data for Ile-de-France ewe lambs and adult ewes under seasonal-transition field conditions, some limitations should be acknowledged. The study was primarily designed to evaluate terminal reproductive outcomes under farm conditions, including oestrus response, pregnancy rate following oestrus, overall pregnancy rate, lambing performance, birth type, litter size, and fecundity. Therefore, endocrine profiles such as progesterone, oestradiol, FSH, and LH concentrations, as well as follicular development dynamics, ovulation rate, corpus luteum characteristics, and embryo quality, were not assessed. For this reason, the observed differences among groups should be interpreted as phenotypic and field-level reproductive responses rather than direct evidence of specific endocrine or ovarian mechanisms. Although parity was balanced between the adult ewe treatment groups through stratified randomization, parity was not evaluated as an independent factor. Postpartum interval was not evaluated as an independent factor and should therefore be considered when interpreting reproductive responses in adult ewes. The natural mating design also represents a limitation of the study. Although rams were randomly assigned to experimental groups and the same ram-to-ewe ratio was maintained across groups, individual ram effects could not be evaluated because individual ewe-to-ram mating assignments were not available in this natural mating design. In addition, because each experimental group was maintained in a separate paddock, a potential paddock effect could not be evaluated independently from the group effect. Accordingly, because potential ram and paddock effects could not be separated from the experimental-group structure, treatment-specific differences should be interpreted with appropriate caution under the present field conditions. In addition, the inclusion of only ewe lambs with previously documented behavioural oestrus, together with the single-breed, single-farm design and defined seasonal-transition conditions, may limit the generalizability of the findings to unselected ewe-lamb populations and to other breeds, management systems, or reproductive seasons. Testing multiple outcomes without adjustment in the primary analyses may increase the risk of Type I error. In the Benjamini–Hochberg FDR sensitivity analysis, the age effects on oestrus onset, overall pregnancy rate, pregnancy rate following oestrus, and fecundity, as well as the dose effect on oestrus onset, remained robust, whereas the dose effects on litter size and multiple birth rate, as well as the age effect on perinatal lamb loss, were more sensitive to multiple-testing adjustment and should therefore be interpreted with caution. Stillbirth and early neonatal mortality were recorded as a single perinatal loss category in the farm records and could not be analysed separately, and perinatal lamb loss was analysed at the lamb level without adjustment for within-litter clustering. Future studies combining reproductive outcomes with serial ultrasonographic monitoring, hormonal measurements, and early embryonic assessments would provide a more comprehensive understanding of the physiological mechanisms underlying age- and dose-related responses to eCG treatment. Although these limitations may affect the interpretation and generalizability of the findings, they did not substantially limit the evaluation of the main objective under the field conditions of this study.

## 5. Conclusions

Under the seasonal-transition field conditions of this study, 500 IU eCG was associated with an earlier onset of oestrus, a higher multiple birth rate and a larger litter size than 400 IU, whereas oestrus, pregnancy and lambing rates were comparable between doses. In the Benjamini–Hochberg FDR sensitivity analysis, the earlier oestrus onset remained robust, whereas the dose effects on multiple birth rate and litter size, as well as the age effect on perinatal lamb loss, were sensitive to adjustment and should therefore be interpreted with caution. Age category was associated with a different set of outcomes: adult ewes reached oestrus earlier and had higher pregnancy rates and fecundity than ewe lambs. The hypothesis that the reproductive response to eCG dose would differ between age categories was not supported, as no significant age × dose interaction was detected. Perinatal lamb loss did not differ between doses but was lower in adult ewes than in ewe lambs, and 91.2% of lambs born were alive at 24 h overall. For practical application, 500 IU eCG may be considered where the objective is earlier oestrus or greater prolificacy, whereas no advantage of 500 IU over 400 IU was detected for pregnancy establishment.

## Figures and Tables

**Table 1:** Reproductive parameters evaluated in the present study, including their calculation and unit of expression.

Parameter	Calculation	Unit
Oestrus rate	Number of animals exhibiting oestrus/total number of animals × 100	%
Oestrus onset	Interval between vaginal sponge removal and the first observed oestrus	h
Overall pregnancy rate	Number of animals diagnosed pregnant by ultrasonography/all synchronized animals × 100	%
Pregnancy rate following oestrus	Number of animals diagnosed pregnant/animals exhibiting oestrus × 100	%
Lambing rate	Number of animals that lambed/animals diagnosed pregnant × 100	%
Pregnancy loss after pregnancy diagnosis	Number of animals diagnosed pregnant but that did not lamb/animals diagnosed pregnant × 100	%
Single birth rate	Number of animals with a single birth/animals that lambed × 100	%
Multiple birth rate	Number of animals with twins or more lambs/animals that lambed × 100	%
Litter size	Total number of lambs born/number of ewes that lambed	lambs/ewe
Fecundity	Total number of lambs born/number of ewes exhibiting oestrus	lambs/ewe
Perinatal lamb loss	Number of lambs born dead or dying within the first 24 h after birth/total number of lambs born × 100	%
Lamb survival to 24 h	Number of lambs alive at 24 h after birth/total number of lambs born × 100	%

Pregnancy loss after pregnancy diagnosis is mathematically complementary to lambing rate and was reported descriptively only.

**Table 2:** Reproductive outcomes (%) of ewe lambs and adult ewes treated with different doses of eCG.

	EL400	EL500	AE400	AE500	*p*-Value
Oestrus rate	73.98 (128/173)	76.33 (129/169)	74.75 (225/301)	77.12 (209/271)	0.861
Overall pregnancy rate	57.23 (99/173)	53.25 (90/169)	62.46 (188/301)	65.68 (178/271)	0.046
Pregnancy rate following oestrus	77.34 (99/128)	69.77 (90/129)	83.56 (188/225)	85.17 (178/209)	0.002
Lambing rate	95.96 (95/99)	98.89 (89/90)	98.40 (185/188)	94.94 (169/178)	0.157
Pregnancy loss after pregnancy diagnosis	4.04 (4/99)	1.11 (1/90)	1.60 (3/188)	5.06 (9/178)	-
Single birth rate	56.84 (54/95)	58.43 (52/89)	63.78 (118/185)	49.11 (83/169)	0.049
Multiple birth rate	43.16 (41/95)	41.57 (37/89)	36.22 (67/185)	50.89 (86/169)	0.049
Perinatal lamb loss	12.41 (17/137)	11.11 (14/126)	7.00 (18/257)	7.63 (20/262)	0.210
Lamb survival to 24 h	87.59 (120/137)	88.89 (112/126)	93.00 (239/257)	92.37 (242/262)	-

Values are expressed as percentages (*n/N*). For each parameter, *n/N* represents the numerator and denominator used for that specific calculation. Denominators varied according to the sequential reproductive stage evaluated: oestrus rate and overall pregnancy rate were calculated using all synchronized animals in each group; pregnancy rate following oestrus was calculated using animals that exhibited oestrus; lambing rate and pregnancy loss after pregnancy diagnosis were calculated using animals diagnosed as pregnant; single and multiple birth rates were calculated using animals that lambed. EL400 and EL500 represent ewe lambs, and AE400 and AE500 represent adult ewes treated with 400 or 500 IU eCG. *p*-values represent omnibus chi-square comparisons among the four experimental groups. Pregnancy loss after pregnancy diagnosis is mathematically complementary to lambing rate and was therefore reported descriptively rather than analyzed as an independent inferential outcome. Perinatal lamb loss was calculated using all lambs born in each group. Lamb survival to 24 h is mathematically complementary to perinatal lamb loss and was therefore reported descriptively rather than analyzed as an independent inferential outcome.

**Table 3:** Effect estimates, 95% confidence intervals, and *p*-values from the 2 × 2 factorial analyses of age category, eCG dose, and their interaction for the evaluated reproductive parameters.

Parameter	Age Category	eCG Dose	Age × Dose
Oestrus rate	OR = 1.04 (0.76–1.42); *p* = 0.792	OR = 1.14 (0.84–1.54); *p* = 0.406	OR = 1.00 (0.54–1.87); *p* = 0.989
Interval to oestrus onset (h)	**Δ = −7.16 (−8.67, −5.64); *p* < 0.001**	**Δ = −2.64 (−4.10, −1.17); *p* < 0.001**	Δ = +0.90 (−2.12, +3.92); *p* = 0.559
Overall pregnancy rate	**OR = 1.44 (1.09–1.89); *p* = 0.009**	OR = 1.02 (0.78–1.34); *p* = 0.870	OR = 1.35 (0.78–2.33); *p* = 0.281
Pregnancy rate following oestrus	**OR = 1.94 (1.33–2.83); *p* = 0.001**	OR = 0.89 (0.61–1.30); *p* = 0.547	OR = 1.67 (0.78–3.59); *p* = 0.186
Lambing rate	OR = 0.80 (0.28–2.32); *p* = 0.684	OR = 0.65 (0.24–1.72); *p* = 0.379	OR = 0.08 (0.01–1.07); *p* = 0.064
Single/multiple birth rate	OR = 1.04 (0.72–1.49); *p* = 0.843	**OR = 1.45 (1.03–2.04); *p* = 0.033**	OR = 1.95 (0.94–4.01); *p* = 0.071
Litter size	Δ = +0.04 (−0.06, +0.13); *p* = 0.448	**Δ = +0.10 (0.01, +0.19); *p* = 0.039**	Δ = +0.19 (−0.01, +0.38); *p* = 0.058
Fecundity	**Δ = +0.17 (0.05, +0.29); *p* = 0.004**	Δ = +0.04 (−0.08, +0.15); *p* = 0.548	Δ = +0.20 (−0.03, +0.44); *p* = 0.090
Perinatal lamb loss	**OR = 0.59 (0.36–0.97); *p* = 0.039**	OR = 1.00 (0.61–1.64); *p* = 0.993	OR = 1.24 (0.46–3.39); *p* = 0.670

OR: odds ratio for binary outcomes (main-effect comparisons: Adult ewe vs. Ewe lamb; 500 IU vs. 400 IU). Δ: mean difference in original units (Adult ewe − Ewe lamb; 500 IU − 400 IU). Age × Dose estimates represent the interaction term. All estimates are presented with 95% confidence intervals. For the age × dose interaction in lambing rate, the OR and 95% CI were obtained from the standard factorial logistic regression model (as for all other estimates), whereas the *p*-value was derived from a Monte Carlo permutation test (Python), given the small number of non-lambing events. Bold values indicate statistical significance (*p* < 0.05).

**Table 4:** Odds ratios (95% confidence intervals) for the key binary reproductive outcomes with a significant single main effect (age category or eCG dose) in the factorial analysis.

Parameter	Comparison	Odds Ratio	95% CI
Overall pregnancy rate	Adult ewe vs. Ewe lamb	1.44	1.09–1.89
Pregnancy rate following oestrus	Adult ewe vs. Ewe lamb	1.94	1.33–2.83
Multiple birth rate	500 IU vs. 400 IU	1.45	1.03–2.04
Perinatal lamb loss	Adult ewe vs. Ewe lamb	0.59	0.36–0.97

Continuous outcomes with a significant main effect (interval to oestrus onset, litter size, fecundity) are not included, as odds ratios do not apply to continuous variables. These are reported as group means in Figure 2.

## Data Availability

The original contributions presented in this study are included in the article. Further inquiries can be directed to the corresponding author.

## Abbreviations

The following abbreviations are used in this manuscript:

eCG	Equine Chorionic Gonadotropin
EL	Ewe Lamb
AE	Adult Ewe
SPSS	Statistical Package for the Social Sciences
SEM	Standard Error of the Mean
FSH	Follicle-Stimulating Hormone
LH	Luteinizing Hormone
GnRH	Gonadotropin-Releasing Hormone
ORs	Odds ratios
